# Left-right tympanal size asymmetry in the parasitoid fly *Ormia ochracea*

**DOI:** 10.17912/micropub.biology.001243

**Published:** 2024-08-02

**Authors:** Max R. Mikel-Stites, Paul E. Marek, Madeleine E. Hellier, Anne E. Staples

**Affiliations:** 1 Engineering Mechanics Program, Virginia Tech, Blacksburg, Virginia, United States; 2 Department of Mathematics, Virginia Tech, Blacksburg, Virginia, United States; 3 Department of Entomology, Virginia Tech, Blacksburg, Virginia, United States; 4 Department of Biological Sciences, Virginia Tech, Blacksburg, Virginia, United States; 5 Department of Biomedical Engineering and Mechanics, Virginia Tech, Blacksburg, Virginia, United States; 6 Department of Mechanical Engineering, Virginia Tech, Blacksburg, Virginia, United States

## Abstract

*Ormia ochracea* is a parasitoid fly notable for its impressive hearing abilities relative to its small size. Here, we use it as a model organism to investigate if minor size differences in paired sensory organs may be beneficial or neutral to an organism's perception abilities. We took high-resolution images of tympanal organs from 21 *O. ochracea* specimens and found a statistically significant surface area asymmetry (up to 6.88%) between the left and right membranes. Numerical experiments indicated that peak values of key sound localization variables increased with increasing tympanal asymmetry, which may explain features of the limited available physiological data.

**Figure 1.**
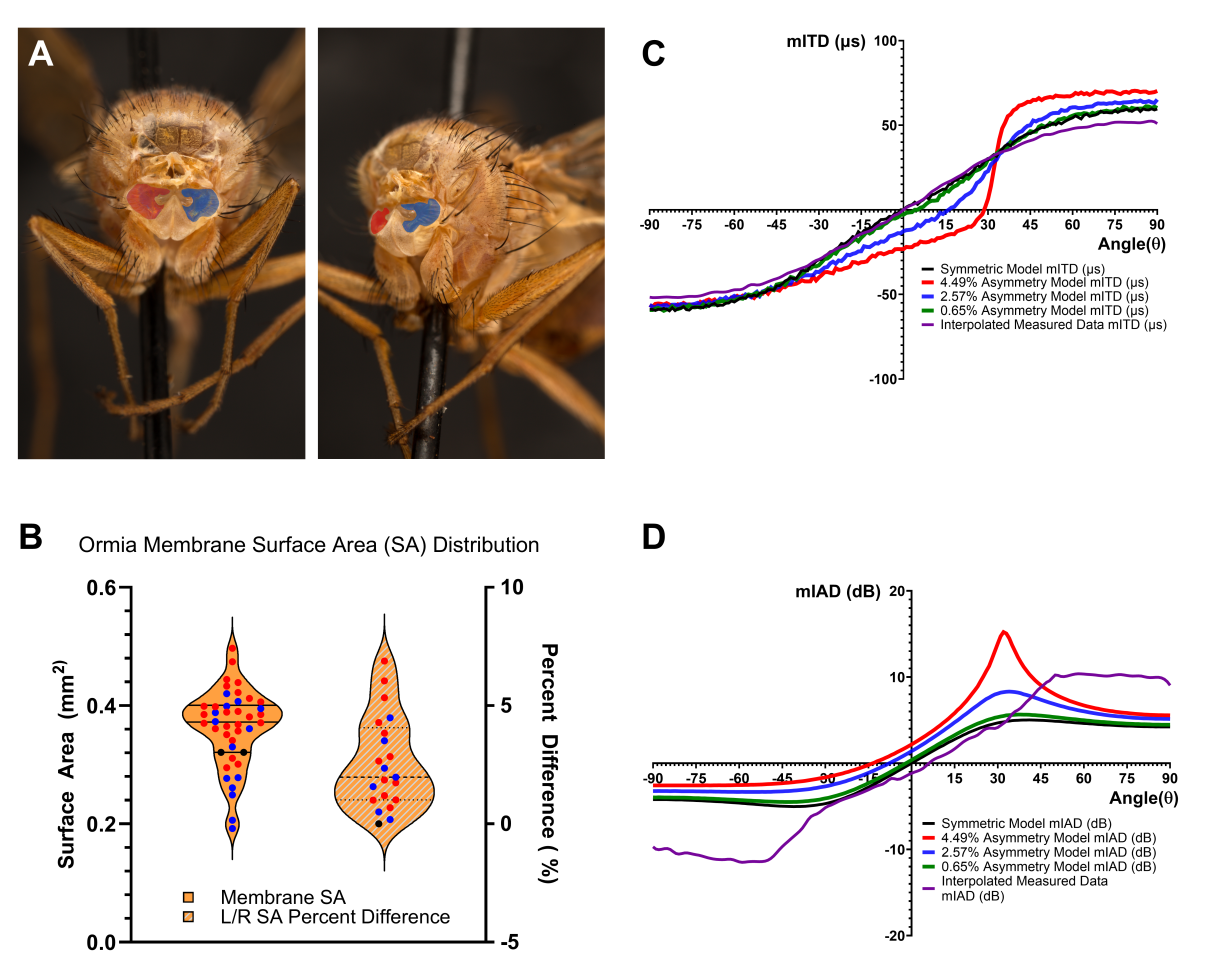
(A) A headless *O. ochracea* fly with left (blue) and right (red) tympanal membranes highlighted. (B) The individual tympanal surface area measurements from Table 1 are shown in the violin plot on the left and the percent difference (using total specimen tympanal surface area in the denominator) between left and right tympanal membrane surface areas measured in the 21 *O. ochracea* specimens are shown in the violin plot on the right. In both plots, data points from flies with a larger left tympanal membrane are blue and those from flies with a larger right tympanal membrane are red. Black data points indicate data from the one symmetric fly in the study. The horizontal lines in the violin plots are quartile lines. (C) Mechanical interaural time difference (mITD) responses for measured and simulated tympanal organs at 5 different levels of asymmetry (none, mean, 1 standard deviation above the mean, and one standard deviation below the mean), as a function of incident sound angle, 𝜃. (D) Mechanical interaural amplitude difference (mIAD) responses for measured and simulated tympanal organs at the same five amounts of surface area asymmetry, as a function of incident sound angle, 𝜃. In the simulation results shown in C and D, the flies are ’right-eared’, that is, the right tympanal membrane is larger than the left. If the left membrane were larger, the change in system response would be mirrored around the x- and y-axes. Additionally, the incident sound used in the simulations was a sinusoid with a frequency of 6 kHz.

## Description

*Ormia ochracea* is a parasitoid fly endemic to the Americas whose gravid females respond phonotactically to calls of their male Gryllidae cricket hosts (Cade, 1975; Walker, 1993) . Surprisingly, *O. ochracea* can locate their hosts with an azimuthal precision of 2 **°** (Mason, 2001; Mason, 2005) --- equal to that of humans --- despite their small size, which should prohibit this level of accuracy because of fundamental constraints imposed by the physics of sound propagation (Mason, 2001; Mikel-Stites, 2023; Rayleigh, 1907) . Miles et al. demonstrated that the fly's two tympanal membranes (highlighted in Figure 1A ) are mechanically coupled, which increases the perceived (or mechanical) interaural time delay and interaural amplitude difference (mITD and mIAD) between the tympana, allowing the fly to resolve nanosecond time differences and greatly increase the precision with which the fly can locate her larval hosts (Miles, 1995; Robert. 1994) as well as avoid predators (Rosen, 2009) . *O. ochracea* is unusual not only in its hearing abilities but also in that its hearing is well-represented by a simple analytical model. Given the ubiquity of small asymmetries in the size of bilateral animals' sensory organs, we chose *O. ochracea* as a model organism to test the hypothesis that a small asymmetry in bilateral sensory organ size could benefit, or at least not harm, an organism's sensing abilities. Here, we present the first measurements documenting tympanal size asymmetry in *O. ochracea* . Additionally, we use an established mathematical model of hearing in *O. ochracea* to predict the impact of the measured asymmetry on binaural auditory cues important for sound localization, the mechanical interaural time delay (mITD) and the mechanical interaural amplitude difference (mIAD).

The tympanal organ of *O. ochracea* extends forward from underneath the fly's cervix (refer to Figure 1A ). Lateral surfaces on the organ, the prosternal tympanal membranes, connect with sharply angled intertympanal bridge arms (Mikel-Stites, 2023) . The organ is a modified structure of the prosternum, and consists of a pair of anteriorly-facing tympanal membranes that cover a hollow cavity within the prosternum. This cavity is exposed to the external environment through tracheal openings connected to the lateral mesothoracic spiracles (Robert, 1994) . Each bridge arm includes a pit that links to an auditory apodeme, which extends longitudinally through the sternal cavity to an auditory sensory organ known as the bulba acustica. The bulba acustica is a chordotonal organ comprising numerous sensory scolopidia. Vibrations are transmitted via the apodemes to the bulbae acusticae, which are connected to the frontal nerve of the thoracic ganglion.

The measurements reported here demonstrated a mean left-right asymmetry in tympanal surface area in our *O. ochracea* population of approximately 2.57% (P=0.0487; see Figure 1B ). A linear regression analysis showed a strong positive linear relationship between the left and right eardrum surface area measurements, with a slope of 1.147 (p-value < 0.0001), indicating that for every 1 mm² increase in the left eardrum surface area, the right eardrum surface area increases by approximately 1.147 mm² on average. An established mathematical model of hearing in *O. ochracea* was modified to incorporate tympanal asymmetry. The model showed that even this small mean asymmetry could have a sizable impact on the peak values of mITD and its spatial distribution (see Figure 1C ). The model also showed a smaller but still noticeable impact on mIAD compared to the symmetric case (see Figure 1D ). When symmetrical tympanal surface area values are used in the model, the resulting mITD and mIAD predictions are in good agreement with the mITD and mIAD values found from physiological measurements made in a sacrificed specimen as reported in Miles et al. (digitized from Figs. 10 and 11 in (Miles, 1995) ), for incoming sound at angles less than 30 **°** from the fly's midline, but the measured and predicted mIAD values diverge for higher incoming sound angles. When tympanal asymmetry is introduced into the model, the predicted mITD values diverge from the measured results, with the difference increasing with both percent tympanal asymmetry and distance from the fly's midline (see Figure 1C ). The predicted mIAD values, though diverging from Miles et al.'s measured values for low and high incoming sound angles, provide a better match to this data for positive intermediate incoming sound angles between approximately 35 **°** and 50 **°** from the fly's midline, for low degrees of right-sided asymmetry (see Figure 1D ). It should be noted that organismal studies have used either single fly specimens or averaged the results of multiple specimens together. In some of these cases asymmetries in responses appear to be present for singular specimens (Miles, 1995; Robert, 1996) . In cases with multiple specimens, it's possible that any present asymmetry was washed out in the averaging process, resulting in apparently mostly-symmetrical responses. This is, unfortunately, an issue that cannot be effectively answered without individual measurement of each specimen's individual physical parameters along with measurement of their biomechanical system responses, which while significantly more time-consuming and arduous, may be the most direct next step to addressing the question of asymmetry more conclusively.

In conclusion, we report a new finding of a small but statistically significant size difference in the tympanal membranes of *O. ochracea* , a well-studied organismal model for binaural hearing (Robert, 1992; Akcakaya, Rosen, Robert, 1994; Miles, 1995; Cade, 1996; Robert, 1998; Robert, 2000; Oshinsky, 2002) . Predictions of the primary cues used in binaural sound localization, the interaural time delay (mITD) and the interauaral amplitude difference (mIAD), made using an established mathematical model of hearing in *O. ochracea* , exhibit increasing peak mITD and mIAD values with increasing tympanal asymmetry, which may indicate enhanced sound localization performance for some incoming sounds angles. However, the model predicts an increasingly poor match with the sole *O. ochracea* mITD/mIAD tympanal response mathematical model validation data set available (Miles, 1995; also analyzed in Robert, 1994) with increasing tympanal asymmetry for most incoming sound angles. We note that this sole validation dataset appears to be obtained from a single sacrificed *O. ochracea* specimen with its head removed. If the fly used to obtain the validation dataset had symmetric tympanal membranes, then this divergence is to be expected.

Furthermore, even if the mathematical model's predictions were highly accurate, the model outputs, mIAD and mITD, merely serve as inputs to the organism's neurological system where additional processing takes place to identify the direction of a sound's source. Whether *O. ochracea* 's now documented tympanal asymmetry is beneficial, neutral, or harmful to its sound localization abilities is unclear. Further physiological measurements with cohorts of flies with varying levels of tympanal asymmetry must be carried out in order to determine whether sound localization in *O. ochracea* is robust to, helped, or harmed by their tympanal asymmetry.

## Methods

Tympanal Measurements

To measure tympanal surface asymmetry in *O. ochracea* , we made 42 separate tympanal measurements of the left and right tympana of 21 specimens obtained from the Virginia Tech Insect Collection and the Smithsonian Museum of Natural History. The specimens were decapitated by hand using sharp-edged tweezers, exposing the tympanal organ, and mounted using a combination of pins and clay to ensure that membranes were perpendicular to the observer. Once mounted, the specimens were photographed with a high-resolution camera (Canon 6D DSLR camera with a 65mm MP-E macro lens). Image stacks were processed digitally to ensure the membranes were in focus, and the tympanal areas were measured using ImageJ analysis software.

Statistical Analysis

**Table 1.** Tympanal asymmetry measurements. Measured data from 21 *O. ochracea* specimens. The first column contains the specimen identifiers, columns two and three contain the left and right surface area measurements in mm ^2^ , and column four contains the absolute differences between the values in columns two and three.

Specimen Identifier, Left Membrane Surface Area (mm ^2^ ), Right Membrane Surface Area (mm ^2^ ), Absolute Surface Area Difference (mm ^2^ )

**Table:** 

USNM01540903, 0.206, 0.192, 0.014
USNM01540904, 0.357, 0.389, 0.032
USNM01540905, 0.261, 0.249, 0.012
USNM01540906, 0.278, 0.277, 0.001
USNM01540907, 0.368, 0.381, 0.013
USNM01540908, 0.361, 0.330, 0.031
USNM01540909, 0.385, 0.406, 0.021
USNM01540910, 0.420, 0.407, 0.013
USNM01540911, 0.341, 0.361, 0.020
USNM01540913, 0.388, 0.373, 0.015
USNM01540914, 0.390, 0.398, 0.008
USNM01540915, 0.439, 0.474, 0.035
USNM01540918, 0.399, 0.444, 0.045
USNM01540920, 0.433, 0.497, 0.064
USNM01540921, 0.295, 0.301, 0.006
USNM01540922, 0.412, 0.422, 0.010
USNM01540923, 0.311, 0.351, 0.040
USNM01540924, 0.399, 0.395, 0.004
USNM01540925, 0.371, 0.385, 0.014
USNM01540919, 0.365, 0.370, 0.005
USNM01540926, 0.321, 0.321, 0.000

Differences were calculated by subtracting the surface area measurements of the left and right tympanal from each other for each *O. ochracea* specimen, producing 21 total difference results. The absolute values of the difference values were then used to determine the percentile differences in left and right by dividing each difference value by each specimen's total tympanal surface areas. The sample standard deviation was calculated for the difference in surface areas and this was then converted to a percentage of the total surface area for each specimen. Figure 1C shows the resulting distribution within the sample population .

A two-tailed paired T-test was used in conjunction with a Pearson correlation coefficient to determine the statistical significance of the results. In the case of the paired T-Test, the left and right surface area values were compared with the standard null hypothesis assumption (that any difference between the left and right was not statistically significant). The resulting p-value was 0.0487, indicating rejection of the null hypothesis and leading to the conclusion that the difference in surface areas was significant. The Pearson correlation coefficient was used to look for correlations between the size of left and right tympanal membranes compared to the overall size (summed surface area of the membranes). The left side had a weak correlation in surface area with overall size (PCC=0.358), and the right had a moderate correlation (PCC=0.527), indicating, at best, a correlation between a larger total surface area and larger right membranes. Statistical power analysis suggests that with increased samples, the p-value would continue to decrease but that the Pearson correlation coefficient results would likely become uncorrelated with total size (assuming that the flies are not 'right-eared' as a general population or something similar). All recorded data are available in Table 1.

Linear regression analysis was carried out on the individual tympanal surface area measurement values. The analysis showed a strong positive linear relationship between the left and right eardrum surface area measurements, with a y-intercept value of -0.042 (p-value: 0.173) and a slope of 1.147 (p-value < 0.0001). The R-squared value for the linear model was 0.912, indicating that approximately 91.2% of the variance in the right eardrum surface area measurements can be explained by the left eardrum surface area measurements. The F-statistic for the analysis was 196.2, and the p-value for the F-statistic was 1.83×10−11. This extremely low value suggests that the model is statistically significant.

Additionally, a chi-squared test of the null hypothesis that the number of “right-eared” (flies with a bigger right ear) and “left-eared” flies is equal in the population we studied carried out. Although there were 13 right-eared and 7 left-eared flies in the population, the p-value found was 0.180, which suggests that the observed distribution of right-eared and left-eared specimens does not significantly differ from the expected 50-50 distribution.

Mathematical Model of Hearing in *O. Ochracea*

The mathematical model of the tympanal response to incident sound in *O. ochracea* that was used to produce Figure 1C,D is the following two coupled ordinary differential equations .

$$[\begin{array}{cc} k_{1}+k_{3} & k_{3}\\ k_{3} & k_{2}+k_{3} \end{array}]x+[\begin{array}{cc} c_{1}+c_{3} & c_{3}\\ c_{3} & c_{2}+c_{3} \end{array}]\dot{x}+[\begin{array}{cc} m & 0\\ 0 & m \end{array}]x^{¨\;}=f$$,

(1)

where *m* is the effective mass of all the moving parts of the tympanal organ, ***x*** = [ *x*_1_ ( *t* ), *x*_2_ ( *t* )] are the unknown responses (positions) of the right (1) and left (2) tympanal centers, and ***f*** = [ *f*_1_ ( *t* ), *f*_2_ ( *t* )] are the acoustic forces incident at the right and left tympanal membrane centers. This model was introduced as Equation 1 in Miles et al. (Miles, 1995) . Miles et al. derived the model by representing the fly's intertympanal bridge as consisting of two rigid beams connected at a pivot point through a coupling spring with stiffness k _3_ and a dashpot with damping constant c _3_ . In their representation, there are also springs and dashpots located at the ends of the bridge beams. The spring and dashpot at the end of the beam that extends from the pivot point to the center of the right tympanal membrane have spring stiffness k _1_ and dashpot damping constant c _1_ , respectively, and the spring and dashpot at the end of the beam that extends from the pivot point to the center of the left tympanal membrane have spring stiffness k _2_ and dashpot damping constant c _2_ , respectively. See Miles et al. (Miles, 1995) for further details about the model.

## References

[R1] Akcakaya M, Nehorai A (2008). Performance analysis of the Ormia ochracea's coupled ears.. J Acoust Soc Am.

[R2] Cade W. (1975). Acoustically Orienting Parasitoids: Fly Phonotaxis to Cricket Song. Science.

[R3] Cade William H., Ciceran Mark, Murray Anne-Marie (1996). Temporal patterns of parasitoid fly (
*Ormia ochracea*
) attraction to field cricket song (
*Gryllus integer*
). Canadian Journal of Zoology.

[R4] Mason AC, Oshinsky ML, Hoy RR (2001). Hyperacute directional hearing in a microscale auditory system.. Nature.

[R5] Mason AC, Lee N, Oshinsky ML (2005). The start of phonotactic walking in the fly Ormia ochracea: a kinematic study.. J Exp Biol.

[R6] Miles RN, Robert D, Hoy RR (1995). Mechanically coupled ears for directional hearing in the parasitoid fly Ormia ochracea.. J Acoust Soc Am.

[R7] Miles RN, Hoy RR (2006). The development of a biologically-inspired directional microphone for hearing aids.. Audiol Neurootol.

[R8] Mikel-Stites MR, Salcedo MK, Socha JJ, Marek PE, Staples AE (2023). Reconsidering tympanal-acoustic interactions leads to an improved model of auditory acuity in a parasitoid fly.. Bioinspir Biomim.

[R9] Oshinsky ML, Hoy RR (2002). Physiology of the auditory afferents in an acoustic parasitoid fly.. J Neurosci.

[R10] Rayleigh Lord (1907). XII.
*On our perception of sound direction*. The London, Edinburgh, and Dublin Philosophical Magazine and Journal of Science.

[R11] Robert D, Amoroso J, Hoy RR (1992). The evolutionary convergence of hearing in a parasitoid fly and its cricket host.. Science.

[R12] Robert D, Read MP, Hoy RR (1994). The tympanal hearing organ of the parasitoid fly Ormia ochracea (Diptera, Tachinidae, Ormiini).. Cell Tissue Res.

[R13] Robert D, Miles RN, Hoy RR (1996). Directional hearing by mechanical coupling in the parasitoid fly Ormia ochracea.. J Comp Physiol A.

[R14] Robert D., Miles R. N., Hoy R. R. (1998). Tympanal mechanics in the parasitoid fly Ormia ochracea  : intertympanal coupling during mechanical vibration. Journal of Comparative Physiology A: Sensory, Neural, and Behavioral Physiology.

[R15] Robert D, Willi U (2000). The histological architecture of the auditory organs in the parasitoid fly Ormia ochracea.. Cell Tissue Res.

[R16] Rosen MJ, Levin EC, Hoy RR (2009). The cost of assuming the life history of a host: acoustic startle in the parasitoid fly Ormia ochracea.. J Exp Biol.

[R17] Walker Thomas J. (1993). Phonotaxis in femaleOrmia ochracea (Diptera: Tachinidae), a parasitoid of field crickets. Journal of Insect Behavior.

